# Identification of a Live Attenuated Vaccine Candidate for Tularemia Prophylaxis

**DOI:** 10.1371/journal.pone.0061539

**Published:** 2013-04-17

**Authors:** Manish Mahawar, Seham M. Rabadi, Sukalyani Banik, Sally V. Catlett, Dennis W. Metzger, Meenakshi Malik, Chandra Shekhar Bakshi

**Affiliations:** 1 Center for Immunology and Microbial Disease, Albany Medical College, Albany, New York, United States of America; 2 Department of Microbiology and Immunology, New York Medical College, Valhalla, New York, United States of America; 3 Albany College of Pharmacy and Health Sciences, Albany, New York, United States of America; University of Louisville, United States of America

## Abstract

*Francisella tularensis* is the causative agent of a fatal human disease, tularemia. *F. tularensis* was used in bioweapon programs in the past and is now classified as a category A select agent owing to its possible use in bioterror attacks. Despite over a century since its discovery, an effective vaccine is yet to be developed. In this study four transposon insertion mutants of *F. tularensis* live vaccine strain (LVS) in Na/H antiporter (*FTL_0304*), aromatic amino acid transporter (*FTL_0291*), outer membrane protein A (OmpA)-like family protein (*FTL_0325*) and a conserved hypothetical membrane protein gene (*FTL_0057*) were evaluated for their attenuation and protective efficacy against *F. tularensis* SchuS4 strain. All four mutants were 100–1000 fold attenuated for virulence in mice than parental *F. tularensis*. Except for the *FTL_0304*, single intranasal immunization with the other three mutants provided 100% protection in BALB/c mice against intranasal challenge with virulent *F. tularensis* SchuS4. Differences in the protective ability of the *FTL_0325* and *FTL_0304* mutant which failed to provide protection against SchuS4 were investigated further. The results indicated that an early pro-inflammatory response and persistence in host tissues established a protective immunity against *F. tularensis* SchuS4 in the *FTL_0325* immunized mice. No differences were observed in the levels of serum IgG antibodies amongst the two vaccinated groups. Recall response studies demonstrated that splenocytes from the *FTL_0325* mutant immunized mice induced significantly higher levels of IFN-γ and IL-17 cytokines than the *FTL_0304* immunized counterparts indicating development of an effective memory response. Collectively, this study demonstrates that persistence of the vaccine strain together with its ability to induce an early pro-inflammatory innate immune response and strong memory responses can discriminate between successful and failed vaccinations against tularemia. This study describes a live attenuated vaccine which may prove to be an ideal vaccine candidate for prevention of respiratory tularemia.

## Introduction

Tularemia is an acute febrile disease caused by *Francisella tularensis*. Tularemia resulting from respiratory infection with type A *F. tularensis* strains is fatal and may result in 30–60% mortality in untreated cases [1]. *F. tularensis* has long been considered a potential biological weapon due to its ability to cause severe illness upon inhalation and is classified as a Tier 1 category A agent by the CDC based on its possible use as a bioterror agent [2]–[5]. All virulent strains belong to *F. tularensis* subsp. *tularensis* (type A) and *F. tularensis* subsp. *holarctica* (type B), whereas avirulent strains are classified under *F. tularensis* subsp. *novicida* or *F. mediasiatica*. One hundred years since its discovery, attempts to develop an effective vaccine against *F. tularensis* have met with limited success.

Subunit or killed vaccine against tularemia has offered limited protection against fully virulent *F. tularensis* [6]–[9]. The *F. tularensis* live vaccine strain (LVS), a derivative of *F. tularensis* subsp. *holarctica* was developed in the USA from the Russian strain S15 [10]. The LVS thus far has emerged as the best vaccine candidate in rendering protection against virulent strains in human and animal studies. The LVS offered protection against subcutaneous or aerosol challenges with the virulent *F. tularensis* type A strains in human volunteers [11]–[14]. However, due to a lack of knowledge about the cause of its attenuation, residual virulence, and inadequate protection, LVS still remains an experimental vaccine. Recent studies have established that *F. tularensis* LVS possesses potent immunosuppressive properties and an ability to undergo phase variation which alters its protective ability [15]. Collectively, these negative traits associated with *F. tularensis* LVS do not allow its use for mass immunization in its current form.

Further attenuation of LVS by genetic modifications has met with limited success against intranasal (i.n.) challenge with type A strains. We have demonstrated earlier that vaccination with a mutant of *F. tularensis* LVS deficient in iron-containing superoxide dismutase, SodB, could protect 40% of C57BL/6 mice against respiratory challenge with 100 CFU of *F. tularensis* SchuS4 [16]. Immunization with several other attenuated mutants of *F. tularensis* LVS have also been shown to offer protection against *F. tularensis* SchuS4 strain in mice. In majority of these studies, over attenuation of LVS mutants resulted either in a complete failure or 20–40% protection in the immunized mice against an i.n. challenge. However, these mutants were found to be protective when an intradermal (i.d.) challenge route was used [17]–[19]. Thus, a vaccine candidate that is attenuated for virulence but still capable of inducing a potent protective immune response, particularly against the respiratory tularemia caused by *F. tularensis* SchuS4 needs to be developed.

Several previous studies have used attenuated mutants of Type A *F. tularensis* SchuS4 as vaccines and have shown protection against homologous SchuS4 challenge [20], [21]. BALB/c mice when immunized with Δ*FTT1103* mutant protected against i.n. challenge with 95 CFU of wild type SchuS4 [22]. Although a defined mutant in *FTT918* provided protection against i.n. challenge with virulent SchuS4 strain, the mutant itself was too virulent to be used as a vaccine [20]. The SchuS4ΔclpB mutant provided 100% protection in i.d. immunized BALB/c mice against an i.n. challenge [23]. In another study, a low dose immunization with the attenuated mutants of SchuS4 either by i.n. or i.d. routes protected mice from a low dose (10 CFU) i.n. challenge. Contrarily, a high dose i.d. immunization with these mutants did not protect immunized mice against a higher (200 CFU) i.n. challenge dose [24]. The results from these studies are promising and the mutants have the potential to be developed into live attenuated vaccines. However, recent classification of *F. tularensis* type A strains by the CDC and Department of Health and Human Services into Tier 1 select agent category [5] may pose serious regulatory issues for the use of Type A *F. tularensis* mutants as a vaccine.

We utilized transposon mutagenesis technique to generate random insertion mutants of *F. tularensis* LVS and screened these mutants in macrophages for properties that correlate with attenuation of virulence (our unpublished data). We have recently reported the role of a mutant in *FTL_0325* gene of LVS in *F. tularensis* pathogenesis. We have demonstrated that infection of macrophages with the *FTL_0325* mutant results in significantly elevated levels of pro-inflammatory cytokines via enhanced activation of NF-κB [25]. In the present study, in addition to the mutant in *FTL_0325* gene which encodes for an outer membrane protein A (OmpA) like protein, we tested three other transposon insertion mutants of *F. tularensis* LVS in genes loci *FTL_0304* (encoding for Na/H antiporter), *FTL_0291* (encoding for aromatic amino acid transporter) and *FTL_0057* that encodes a conserved hypothetical membrane protein for their vaccine potential against an i.n. challenge with *F. tularensis* SchuS4. This study reports that the identified attenuated mutants of *F. tularensis* LVS are safe and induce protective immunity against lethal i.n. challenge with highly virulent *F. tularensis* SchuS4 strain.

## Materials and Methods

### Ethics Statement

This study was carried out in strict accordance with the recommendations and guidelines of National Council for Research (NCR) for care and use of animals. All the animal experiments were conducted in the centralized Animal Resources Facilities of Albany Medical College and New York Medical College licensed by the USDA and the NYS Department of Health, Division of Laboratories and Research and is accredited by the American Association for the Accreditation of Laboratory Care. The use of animals and protocols were approved by the Institutional Animal Care and Use Committee (IACUC) of Albany Medical College (Protocol Number 10-01003) and New York Medical College (151-2-1211.1H). Mice were administered an anesthetic cocktail consisting of ketamine (5 mg/kg) and xylazine (4 mg/kg) and underwent experimental manipulation only after they failed to exhibit a toe pinch reflex. Although, in time-to-death experiments death was intended to be used as the experimental endpoint, mice exhibiting more than 20% weight loss, anorexia, dehydration and impairment of mobility were removed from the study and euthanized by approved means. Humane endpoints were also necessary for mice which survived at the conclusion of the experiment. Mice were administered an anesthetic cocktail of ketamine and xylazine intraperitoneally (i.p.) and then euthanized via cervical dislocation followed by cardiac puncture, a method that is consistent with Albany Medical College GLP Standard Operating Procedure on “Guidelines for Animal Euthanasia” (Document Number ARF-VC-004) and recommendations of the Panel on Euthanasia of the American Veterinary Medical Association. In all experimental procedures efforts were made to minimize pain and suffering.

### Bacterial Strains

*F. tularensis* LVS (American Type Culture Collection 29684) was provided by Dr. K. Elkins (U.S. Food and Drug Administration, Bethesda). *F. tularensis* SchuS4 was obtained from the U.S. Army Medical Research Institute for Infectious Diseases (Frederick) and BEI Resources (Manassas, VA). All the *Francisella* strains were cultured in Mueller Hinton Broth (MHB) or MH-chocolate agar plates as described previously [26]. Mutants in *FTL_0304*, *FTL_0291*, *FTL_0325* and *FTL_0057* were identified as being deficient for intramacrophage growth in a transposon mutant screen of *F. tularensis* LVS in the alveolar macrophage cell line, MH-S (our unpublished data).

### Growth Curves

The growth characteristics of the *FTL_0325* and *FTL_0304* mutants grown in MHB were evaluated using high throughput assays in a 96-well plate as described earlier [27], [28]. The growth curves in Brain-Heart Infusion broth (BHI) and Chamberlain’s defined medium (CDM) were prepared by growing bacteria in 25 ml culture volume following a protocol described earlier [26]. The optical readings (OD_600_) were recorded at 4 hr intervals and comparisons were made with the growth attributes of wild type *F. tularensis* LVS grown under similar conditions.

### Mouse Studies

All mouse experiments were done in accordance with the approved protocols from Institutional Animal Care and Use Committees of Albany Medical College and New York Medical College. Six to eight week old C57BL/6 and BALB/c mice (Taconic) were anaesthetized prior to inoculation via i.p. injection of a cocktail of ketamine (Fort Dodge Animal Health, Fort Dodge) and xylazine (Phoenix Scientific, St. Joseph). Mice were challenged i.n. with 1×10^6^ or 1×10^7^ CFU of wild type *F. tularensis* LVS or the mutants in 20 µl (10 µl/nare) of PBS. The infected mice were monitored twice daily for morbidity and mortality for a period of 28 days.

The bacterial numbers were quantified in the homogenates of lung, liver and spleen of mice infected with the WT *F. tularensis* LVS, *FTL_0325* or *FTL_0304* mutant at various times post-challenge as described previously [16]. The lungs from infected mice were excised and processed using standard histological procedures. The paraffin embedded sections were stained with Hematoxylin–Eosin (H&E) and examined by light microscopy.

For protection studies, BALB/c mice were immunized i.n. with 1×10^7^ CFU of the mutants and challenged i.n. with 100 CFU of SchuS4 in 20 µl of PBS (10 µl/nare) 30 days post-immunization. Unvaccinated mice were included as controls. The mice were observed for 21 days post-challenge for clinical signs, morbidity, and mortality. Mouse experiments with SchuS4 were carried out in the CDC certified BSL-3/ABSL-3 suite at Albany Medical College.

### Cytokine Measurement

The protein content in the lung and spleen homogenates collected from the infected mice were normalized using a bicinchoninic protein assay kit (Pierce). Mouse Inflammation Cytometric Bead Array flex sets (BD Biosciences) were used for the simultaneous measurement of IFN-γ, TNF-α, IL-6, and MCP-1 as previously described [16].

### Antibody Measurement

Six to eight week old BALB/c mice were immunized with 1×10^7^ CFU of the *FTL_0325* or *FTL_0304* mutants i.n. The mice were bled at days 45 and 60 post-infection (PI), the sera were separated and quantitated for *F. tularensis* specific total IgG levels by ELISA as described previously [26].

### Determination of Recall Responses in Mice Vaccinated with the *FTL_0325* and *FTL_0304* Mutants

A method described earlier [29] was used to determine memory responses in the vaccinated mice. Briefly, BALB/c mice were immunized with 1×10^7^ CFU of the *FTL_0325* or *FTL_0304* mutants by i.n. route. At 45 and 60 days post-immunization, splenocytes were collected from the immunized mice. Based on the differences in the virulence of *F. tularensis* LVS and SchuS4, BMDMs prepared from naive BALB/c mice were infected with 100 MOI of LVS or 20 MOI of *F. tularensis* SchuS4 for two hrs and treated with gentamicin (50 µg/ml) to kill the extracellular bacteria. The infected BMDMs were co-cultured with splenocytes from immunized mice in a ratio of 1∶1 for a period of 48 hrs (*F. tularensis* LVS infected BMDMs) and for 120 hrs (*F. tularensis* SchuS4 infected BMDMs). The culture supernatants were collected at the indicated times and analyzed for IFN-γ and IL-17a by BD-flex sets as described above. The BMDMs infected with *F. tularensis* SchuS4 and co-incubated with splenocytes from naïve, the *FTL_0325* or *FTL_0304* immunized mice were lysed with 0.1% sodium deoxycholate at 72 hrs PI, diluted 10-fold in sterile phosphate buffered saline (PBS) and plated on MH-chocolate agar plates (BD Biosciences) to determine the intracellular bacterial replication. The bacterial colonies were counted after 48 hrs and results were expressed as log_10_ CFU/ml. The experiments with *F. tularensis* SchuS4 were conducted in a CDC-certified BSL-3 laboratory at New York Medical College.

### Statistical Analysis

Results were expressed as mean ± standard error of mean (SEM) or standard deviation (SD). The comparisons between multiple groups were made using One-way Analysis of Variance (ANOVA) with Tukey-Kramer Multiple Comparisons post-test. Differences between the experimental groups were considered statistically significant at a P < 0.05 level. The survival data were analyzed using Logrank test and P-values were determined. Differences in survival between the experimental groups were considered statistically significant at a P < 0.005 level. The correlation between bacterial load and cytokine levels at days 1 and 4 in the lungs and at day 4 in the spleen were determined. The results of correlation analysis were expressed as P-value (for the hypothesis test) and an r^2^ value (for coefficient of determination). An r^2^ value of 0.6 and above was considered as a strong correlation.

## Results

### The *FTL_0304*, *FTL_0291*, *FTL_0325* and *FTL_0057* Mutants of *F. tularensis* LVS are Attenuated for Virulence in Mice

The virulence of all four mutants was tested in C57BL/6 and BALB/c mice. The mice were inoculated i.n. with the mutants and wild type *F. tularensis* LVS, and monitored for a period of 28 days for morbidity and mortality. It was observed that all four mutants were attenuated for virulence as 100% of BALB/c or C57BL/6 survived a dose as high as 1×10^7^ CFU inoculated i.n. On the contrary, 100% of the mice inoculated either with 1×10^6^ or 1×10^7^ CFU of *F. tularensis* LVS succumbed to infection by days 5–7 PI (Table 1). These results demonstrate that *FTL_0291*, *FTL_0057*, *FTL_0304* and *FTL_0325* gene products are required for virulence in mice.

**Table 1:** *FTL_0304*, *FTL_0291*, *FTL_0325* and *FTL_0057* mutants are highly attenuated for virulence in BALB/c and C57BL/6 mice.

Strains	1×10^6^ CFU^a^		1×10^7^ CFU^a^	
	BALB/c	C57BL/6	BALB/c	C57BL/6
*F. tularensis* LVS	0/5	0/5	0/5	0/5
*FTL_0304*	5/5	5/5	5/5	5/5
*FTL_0291*	5/5	5/5	5/5	5/5
*FTL_0325*	5/5	5/5	5/5	5/5
*FTL_0057*	5/5	5/5	5/5	5/5

a 6–8 weeks old mice were infected i.n. with either *F. tularensis* LVS or the indicated mutants and monitored for mortality for 28 days. Data are shown as number of mice survived/total number of mice infected.

### Immunization with *FTL_0291*, *FTL_0325* and *FTL_0057* Protect BALB/c Mice Against *F. tularensis* SchuS4 Challenge

**Figure 1:**
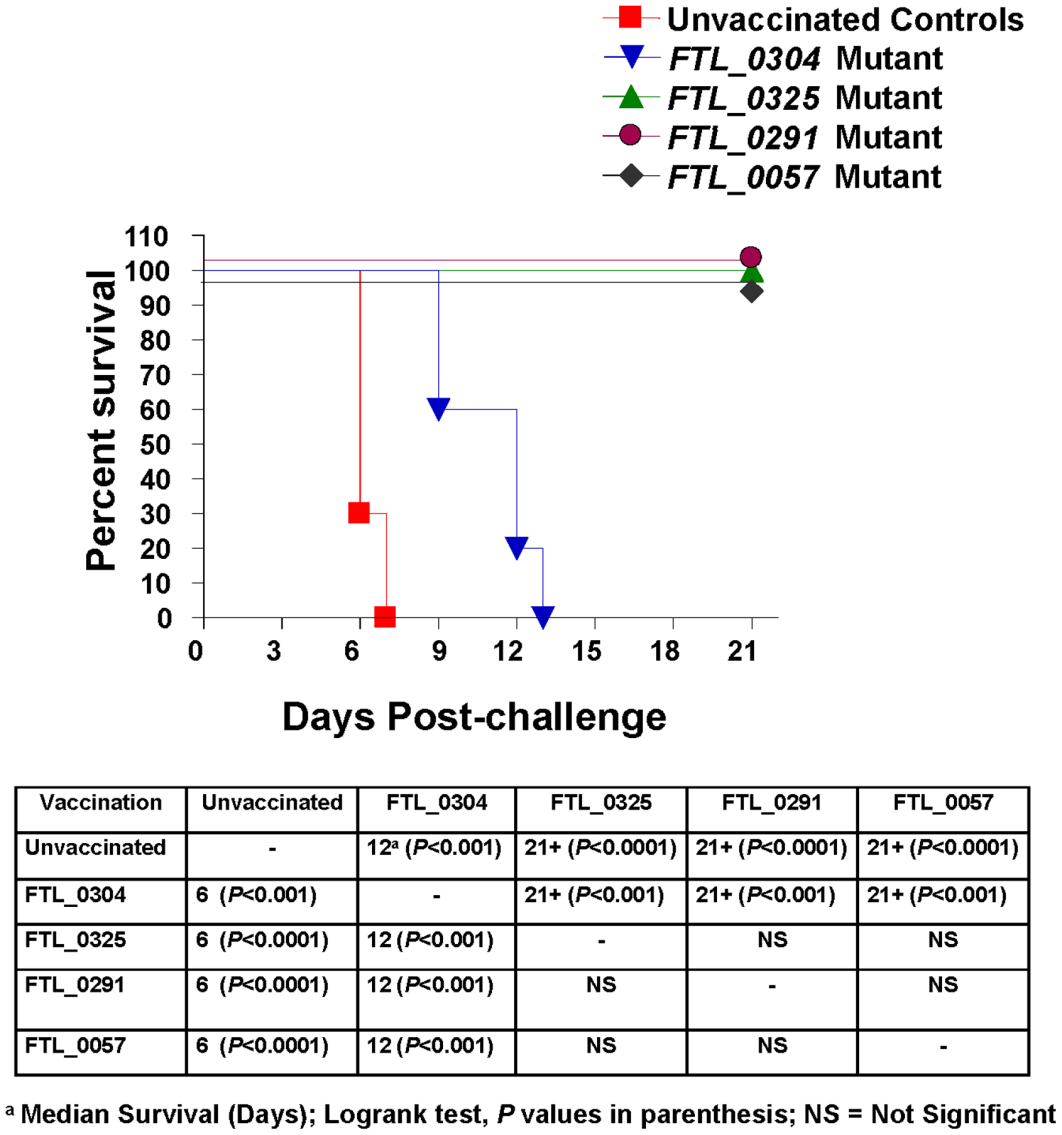
Immunization with the *FTL_0291*, *FTL_0325* or *FTL_0057* mutant protects BALB/c mice against *F. tularensis* SchuS4 challenge. BALB/c mice (n = 10) were immunized i.n. with 1×10^7^ CFU of the indicated mutants, challenged i.n. with 100 CFU of *F. tularensis* SchuS4 30 days post-immunization, and observed for mortality for a period of 21 days. Unvaccinated mice were kept as controls. Data are represented as Kaplan-Meier survival curves and are cumulative of two independent experiments. The *P* values were determined using Logrank test. Survival comparisons between mice immunized with the *FTL_0291*, *FTL_0325* or *FTL_0057* mutant and unvaccinated controls or *FTL_0304* immunized mice are shown in the table. Differences in survival between the experimental groups were considered statistically significant at a *P* < 0.005 level.

A dose as low as 1–10 CFU of *F. tularensis* SchuS4 strain given i.n. can cause mortality in mice within 4–6 days [30]. BALB/c mice were immunized i.n. with 1×10^7^ CFU of the *FTL_0304 FTL_0291*, *FTL_0325*, and the *FTL_0057* mutants. A high immunization dose was chosen to avoid requirement for subsequent boosters and to evaluate the safety and efficacy of a single shot vaccine. The immunized mice were challenged with *F. tularensis* SchuS4 30 days post-immunization. All naïve unvaccinated mice succumbed to a 100 CFU i.n. challenge of SchuS4 with a median survival time of 6 days. Similarly, 100% of mice immunized with the *FTL_0304* mutant succumbed to infection with a median survival time of 12 days which was significantly higher than the naïve unvaccinated controls. A single dose immunization with the *FTL_0291*, *FTL_0325* and *FTL_0057* mutant protected 100% of BALB/c mice against i.n. challenge with 100 CFU of *F. tularensis* SchuS4 and all mice survived for 21 days (Fig. 1). Survival differences between *FTL_0291*, *FTL_0325, FTL_0057* mutants and naïve unvaccinated or *FTL_0304* vaccinated mice were statistically significant when analyzed by Logrank test. These results indicate that the *FTL_0291*, *FTL_0325* and *FTL_0057* mutants retain their antigenic properties and protect the immunized mice, while immunization with the *FTL_0304* mutant fails to protect mice against the *F. tularensis* SchuS4 challenge.

The results from protection studies indicated that the mutants of *F. tularensis* differ in their protective capabilities against an i.n. SchuS4 challenge. We hypothesized that these variations are due to the differences in their ability to persist in the host tissue and to generate an early innate immune response. We chose the *FTL_0325* mutant over *FTL_0291* or *FTL_0057* mutants to investigate its vaccine potential based on its ability to induce an elevated pro-inflammatory innate immune response as we have reported recently [25]. Additionally, the *FTL_0304* mutant that did not protect the immunized mice against SchuS4 challenge was also chosen to understand the differences in protective versus non-protective immune responses. These two mutants were evaluated for their ability to colonize, persist in the host tissue and to induce an inflammatory response.

### The *FTL_0304* and *FTL_0325* Mutants of *F. tularensis* Exhibit no Growth Defect Under Acellular Growth Conditions

**Figure 2:**
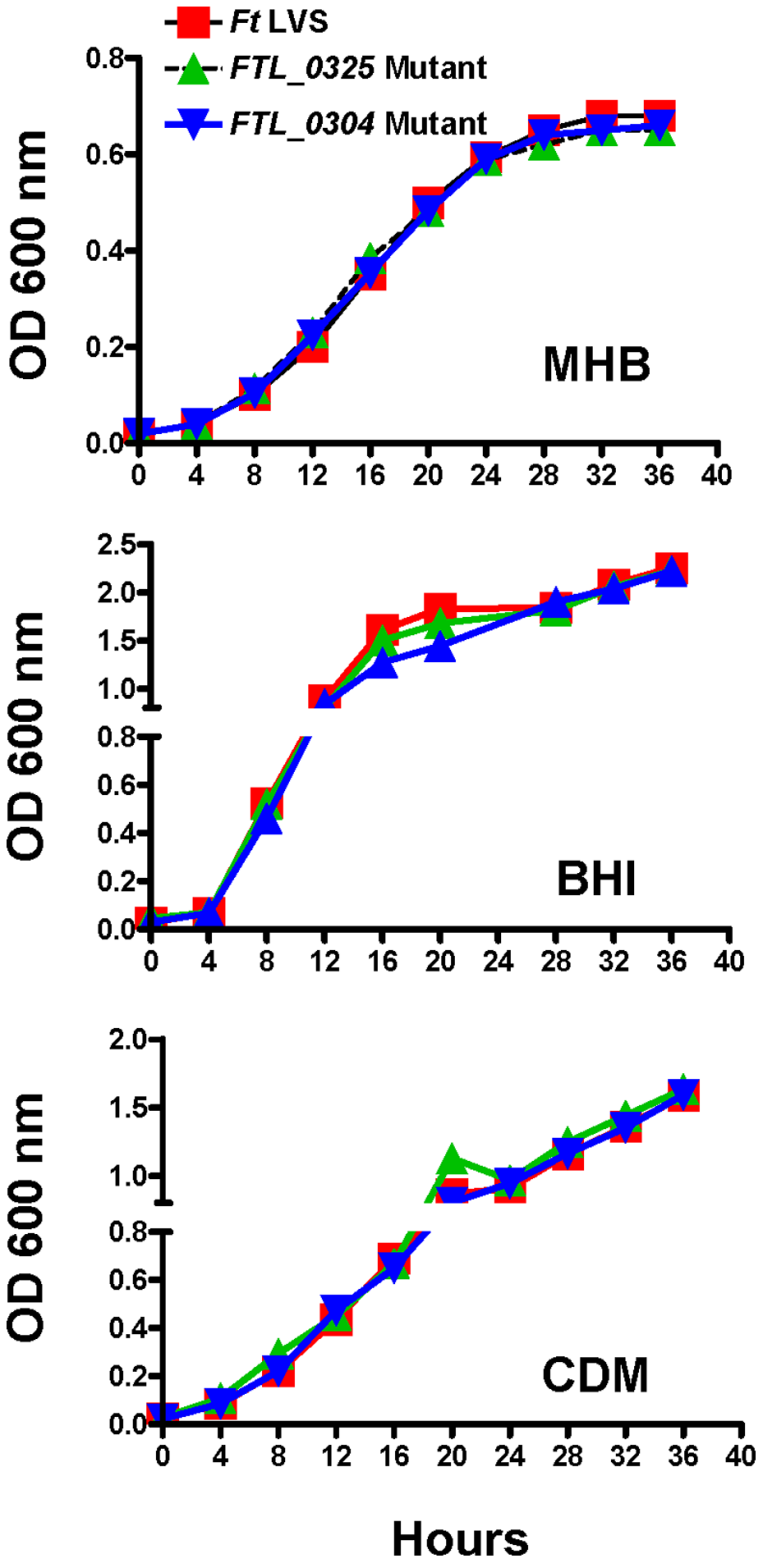
The *FTL_0325* and *FTL_0304* mutants of *F. tularensis* LVS exhibit no growth defect under acellular growth conditions. Growth curves for bacteria grown in MHB were generated in a 96-well plate using 200 µl culture volumes while the growth curves for bacteria grown in BHI and CDM were generated using a 25 ml culture volume. Results shown are representative of two independent experiments.

In an earlier study, we have demonstrated that the *FTL_0304* and *FTL_0325* mutants are attenuated for intramacrophage survival as compared to the wild type *F. tularensis* LVS using a macrophage cell culture invasion assay [25]. In this study, these mutants were further characterized to understand if the intramacrophage growth defects were due to their inability to grow under acellular growth conditions. The mutants and the wild type *F. tularensis* LVS were inoculated in MHB, BHI broth or CDM. The OD at 600 nm was recorded over a period of 36 hrs and the growth curves were generated. It was observed that both the *FTL_0325* and *FTL_0304* mutants grew similar to the *F. tularensis* LVS in all the three growth media tested and did not reveal any growth defect (Fig. 2). These results indicate that the *FTL_0304* and *FTL_0325* mutants are capable of growing under acellular growth conditions and are killed only when exposed to the macrophage environment.

### The *FTL_0325* Mutant Persists Longer than *FTL_0304* Mutant in Immunized Mice

**Figure 3:**
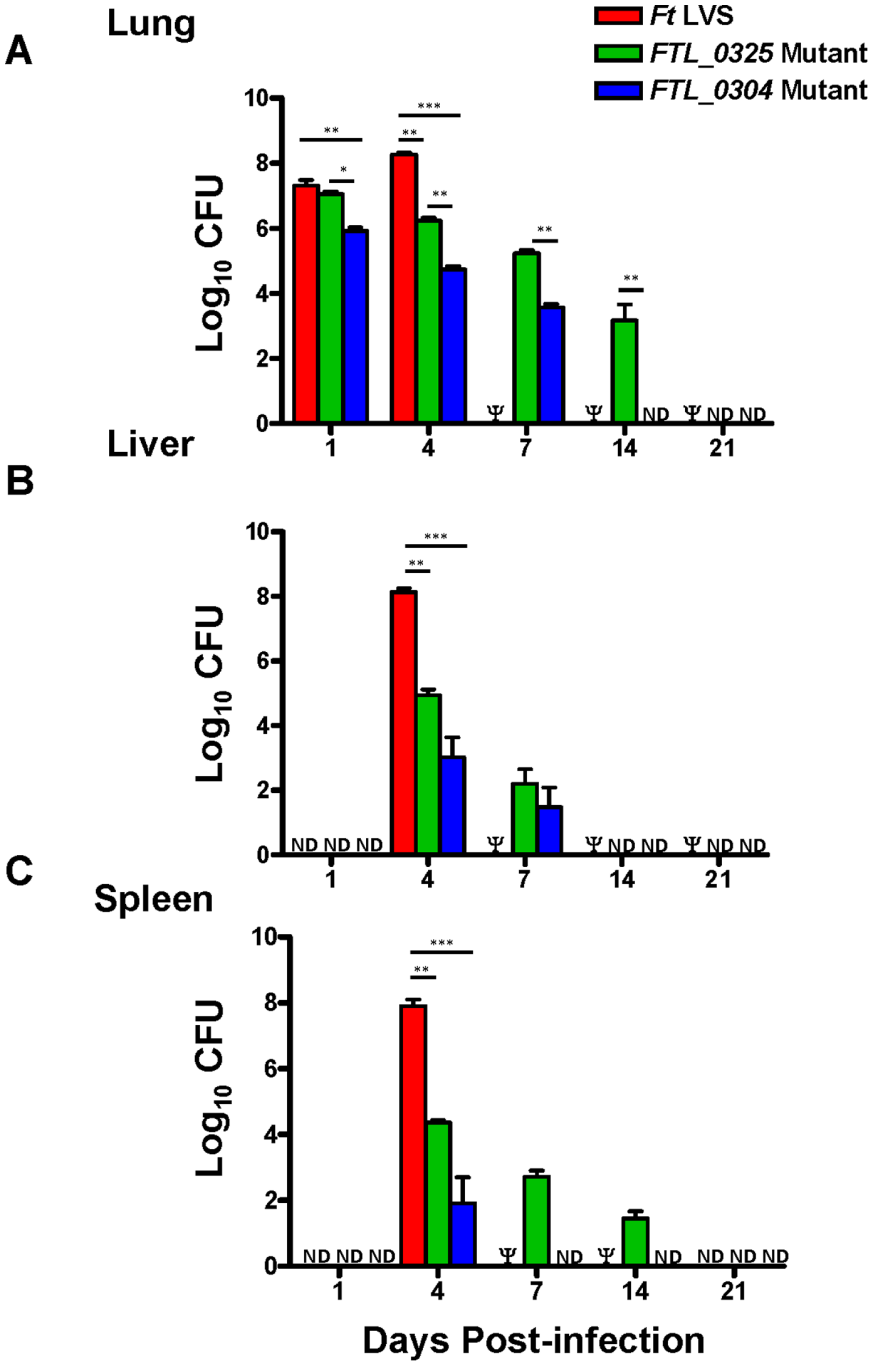
The *FTL_0325* mutant persists longer than the *FTL_0304* mutant in infected mice. BALB/c mice (n = 4) were infected with 1×10^7^ CFU of the *F. tularensis* LVS, *FTL_0325* or *FTL_0304* mutant. At the indicated times, homogenates of lung (**A**), liver (**B**) and spleen (**C**) from the infected mice were diluted 10-fold and plated on MH-chocolate agar plates for quantification of bacterial numbers. The results are expressed as Log_10_ CFU/organ and are representative of two independent experiments conducted. The data were analyzed using ANOVA with Tukey-Kramer Multiple Comparison post-test and *P* values were recorded. *P<0.05; **P<0.01; ***P<0.001. Ψ = Mice infected with 1×10^7^ CFU of *F. tularensis* LVS succumbed to infection by day 7 PI and hence were unavailable for comparison. ND = Not detected.

BALB/c mice were infected i.n. with 1×10^7^ CFU of *F. tularensis* LVS, the *FTL_0304* or *FTL_0325* mutant, sacrificed at days 1, 4, 7, 14 and 21 PI and the bacterial numbers recovered from lungs, liver and spleen were quantitated. Significantly lower numbers of *FTL_0304* mutant were recovered as compared to the wild type *F. tularensis* LVS and the *FTL_0325* mutant from the lungs of infected mice as early as day 1 PI; however, no differences in bacterial numbers between the latter two strains were observed at day 1 PI. The bacterial numbers in *F. tularensis* LVS infected mice increased significantly at day 4 PI and all mice succumbed to infection by day 7 PI (Fig. 3A). The numbers of *FTL_0325* mutant strain were significantly higher than the *FTL_0304* mutant at all the time-points examined; however, their numbers also declined gradually and no bacteria were recovered at day 21 PI. Dissemination of both the mutant strains to liver and spleen occurred similar to the *F. tularensis* LVS when observed at day 4 PI; however, the numbers of *F. tularensis* LVS recovered were significantly higher than those for the two mutants. Similar to the findings in lungs, the numbers of *FTL_0325* mutant were significantly higher than the *FTL_0304* mutant in the liver and spleen (Fig. 3B, C). The *FTL_0304* mutant bacteria were observed in the liver and spleen till day 7 and 4 respectively, while the *FTL_0325* mutant persisted in the spleen until day 14 PI (Fig. 3C). Taken together, these results demonstrate that the *FTL_0304* mutant is highly attenuated for virulence and gets eliminated from the lungs, liver and spleen more rapidly than the *FTL_0325* mutant. These results also demonstrate that the *FTL_0325* mutant persists longer than the *FTL_0304* mutant in infected mice.

### Immunization with the *FTL_0325* Mutant Induces an Early Pro-inflammatory Cytokine Response

**Figure 4:**
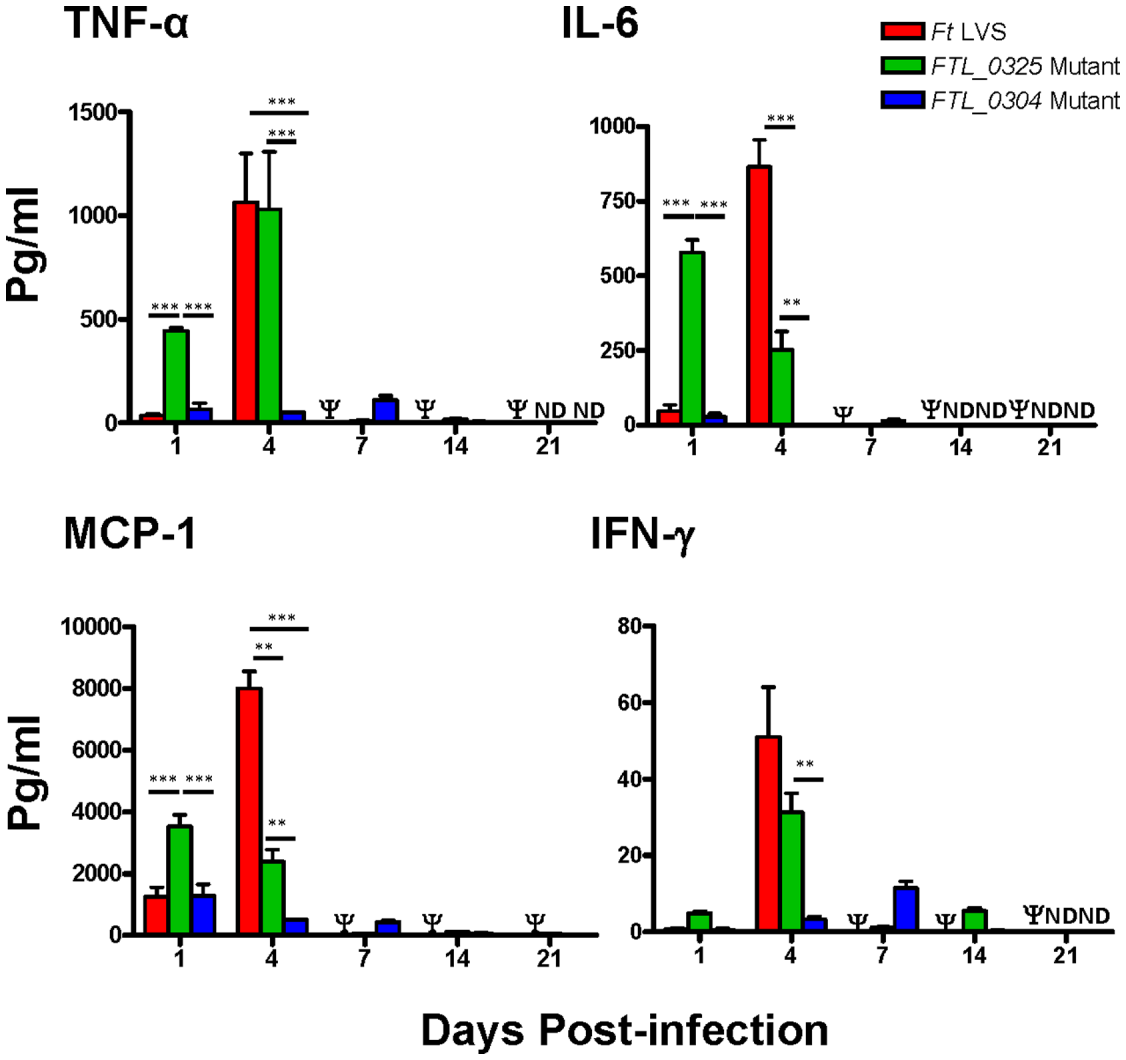
Immunization with the *FTL_0325* mutant induces an early pro-inflammatory cytokine response in lungs. BALB/c mice (n = 4) were infected with 1×10^7^ CFU of the indicated mutants and wild type *F. tularensis* LVS. At the indicated times, the levels of pro-inflammatory cytokines were measured in lung homogenates using a Cytometric Bead Array assay. The data are representative of two independent experiments conducted and were analyzed using ANOVA with Tukey-Kramer Multiple Comparison post-test and *P* values were recorded. **P<0.01; ***P<0.001. Ψ = Mice infected with 1×10^7^ CFU of *F. tularensis* LVS succumbed to infection by day 7 PI and hence were unavailable for comparison.

*F. tularensis* LVS dampens the innate immune response by preventing production of pro-inflammatory cytokines by the innate immune cells. We have recently reported that the macrophages infected with the *FTL_0325* mutant induce significantly higher levels of pro-inflammatory cytokines than those infected either with the *F. tularensis* LVS or the *FTL_0304* mutant [25]. A kinetic experiment was performed to quantitate the levels of pro-inflammatory cytokines TNF-α, IL-6, MCP-1, and IFN-γ in the lung and spleen homogenates of mice infected with *F. tularensis* LVS, *FTL_0325* or *FTL_0304* mutant. Significantly higher levels of TNF-α, IL-6, and MCP-1 were observed in the lungs of *FTL_0325* infected mice at day 1 PI as compared to those infected with equal numbers of wild type *F. tularensis* LVS or the *FTL_0304* mutant (Fig. 4). As the infection progressed, the cytokine profile in the *F. tularensis* LVS infected mice was similar to moribund mice, wherein they exhibit a cytokine storm prior to death [31]. In mice infected with the *FTL_0325* mutant, TNF-α and IFN-γ levels peaked at day-4 PI and then returned to the baseline values. On the contrary, the *FTL_0304* mutant induced extremely lower cytokine levels throughout the course of infection (Fig. 4).

**Figure 5:**
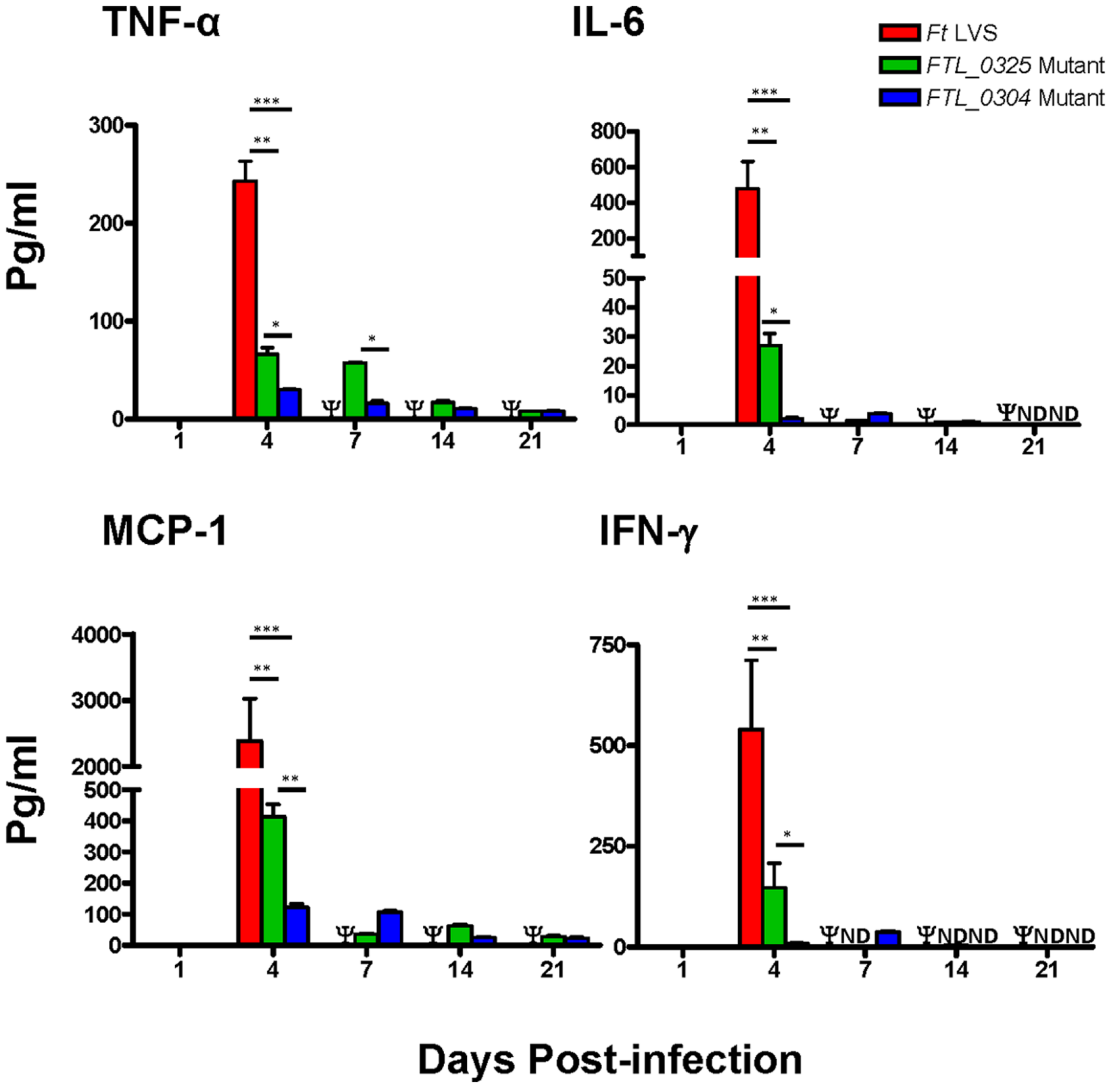
Immunization with the *FTL_0325* mutant induces a higher pro-inflammatory cytokine response in the spleen. BALB/c mice (n = 4) were infected with 1×10^7^ CFU of the indicated mutants and wild type *F. tularensis* LVS. At the indicated times, the levels of pro-inflammatory cytokines were measured in spleen homogenates using a Cytometric Bead Array assay. The data are representative of two independent experiments conducted and were analyzed using ANOVA with Tukey-Kramer Multiple Comparison post-test and *P* values were recorded. *P<0.05; **P<0.01; ***P<0.001. Ψ = Mice infected with 1×10^7^ CFU of *F. tularensis* LVS succumbed to infection by day 7 PI and hence were unavailable for comparison.

Significantly higher levels of TNF-α, IL-6, MCP-1 and IFN-γ were found in the spleens of *FTL_0325* mutant infected mice than the *FTL_0304* mutant infected mice at day 4 PI (Fig. 5). Again, the *F. tularensis* LVS infected mice presented a cytokine profile of moribund mice and the levels of all the cytokines tested were significantly higher than those observed for mice immunized either with the *FTL_0325* or *FTL_0304* mutants. No statistical correlation between bacterial numbers and MCP-1 [0.092 (P<0.33)], TNF-α [0.059 (P<0.44)] and IL-6 [0.104 (P<0.30)] cytokine responses was observed at day 1 PI in the lungs when comparisons were made between the *F. tularensis* LVS, *FTL_0325* and *FTL_0304* mutant infected mice. These results thus reflect the unique ability of the *FTL_0325* mutant to induce an early pro-inflammatory cytokine response. On the other hand, a positive correlation was observed in the lungs and spleens of the *F. tularensis* LVS, *FTL_0325* and *FTL_0304* mutant infected mice at day 4 PI (Table 2). These results indicate that as the infection progresses an increase in bacterial numbers is associated with a corresponding increase in the cytokine responses. Collectively, these results demonstrate that unlike wild type *F. tularensis* LVS and the *FTL_0304* mutant, the *FTL_0325* mutant induces an early and regulated pro-inflammatory cytokine response. These findings further support the role of *FTL_0325* in the modulation of pro-inflammatory cytokine responses.

**Table 2:** Correlation analysis of bacterial burden and pro-inflammatory cytokine levels in mice infected with *F. tularensis* LVS, the *FTL_0325* and *FTL_0304* mutants.

Organ	MCP1	TNF-α	IL-6
Lung D1	0.092* (0.33)**	0.059 (0.44)	0.104 (0.30)
Lung D4	**0.938 (0.000002)**	0.443 (0.018)	**0.912 (0.00001)**
Spleen D4	**0.700 (0.00068)**	**0.905 (0.000001)**	0.590 (0.003)

* r^2^ value of 0.6 and above was considered as a strong correlation. Values in bold are strongly correlated.

** P-value.

### Immunization with the *FTL_0325* Mutant Induces an Early Inflammatory Response at the Site of Infection

**Figure 6:**
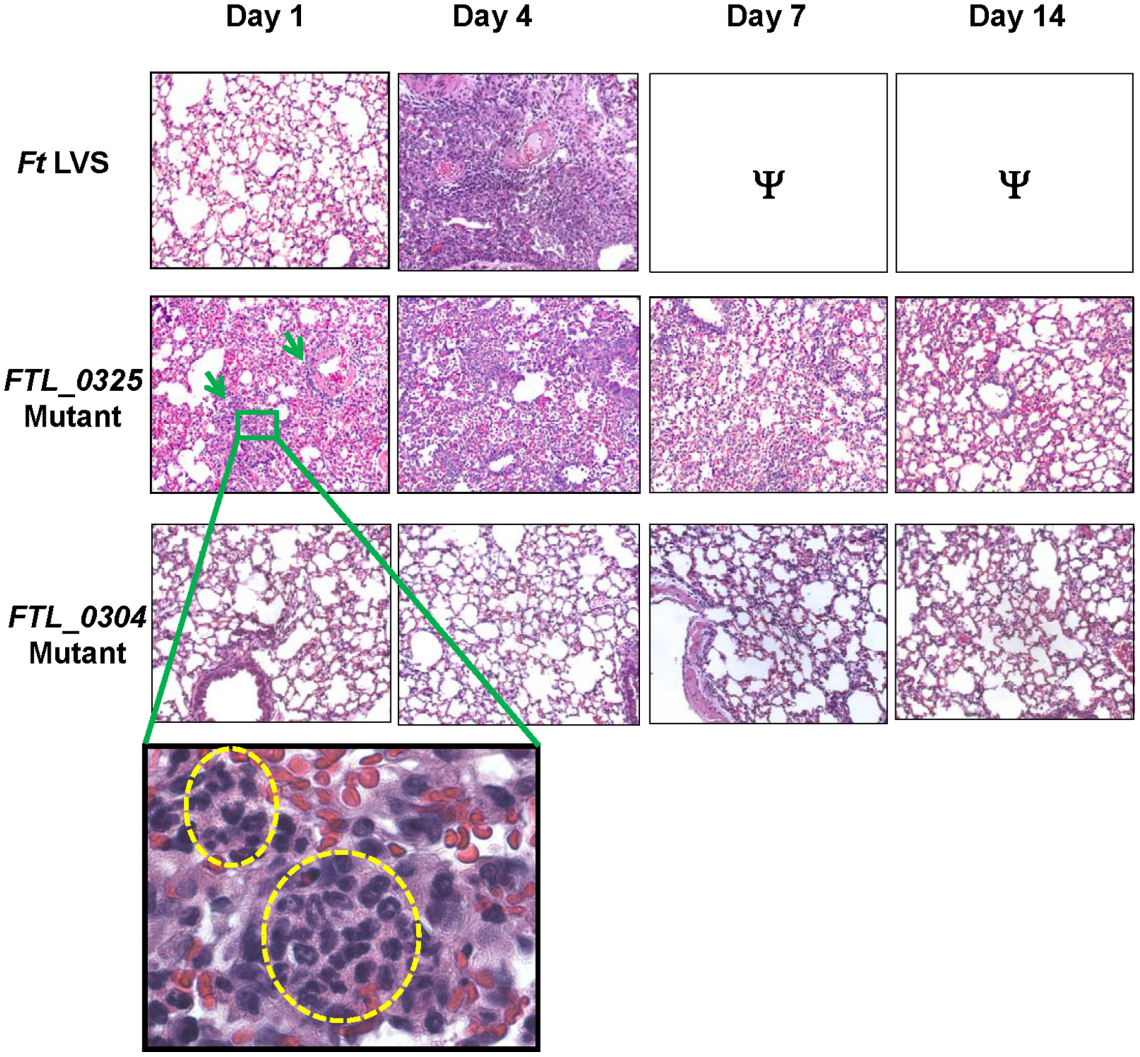
Immunization with the *FTL_0325* mutant induces an early inflammatory response. BALB/c mice were infected with 1×10^7^ CFU of the *F. tularensis* LVS, *FTL_0325* or *FTL_0304* mutant and the lungs were harvested at the indicated times, sectioned and stained with H & E. Representatives of H & E stained lung sections are shown. Infiltration of neutrophils is shown in the inset (Magnification 10×; Inset 100×). Ψ = Mice infected with 1×10^7^ CFU of *F. tularensis* LVS succumbed to infection by day 7 PI and hence were unavailable for comparison. Green arrows indicate the sites of cellular infiltration. Yellow circles in inset show neutrophilic infiltration in the lungs of *FTL_0325* mutant immunized mice.

We next investigated if an early induction of pro-inflammatory cytokines in the mice immunized with the *FTL_0325* mutant also elicits an early inflammatory response in the lungs. We examined the lung sections from mice infected with the *FTL_0325* or *FTL_0304* mutants. Indeed, the H&E stained lung sections from the *FTL_0325* mutant-immunized mice revealed inflammatory foci consisting mostly of neutrophils as early as day 1 PI (Fig. 6 and inset). The cellular infiltration and the numbers of inflammatory foci increased at day 4 PI in the lungs of *FTL_0325* mutant infected mice, which subsided gradually by day 14 PI. In contrast, the lungs of *F. tularensis* LVS and *FTL_0304* infected mice did not exhibit any signs of cellular infiltration at day 1 PI; however, a severe inflammatory response was observed in the lungs of *F. tularensis* LVS infected mice at day 4 PI. Minimal to no cellular infiltration was observed in the lungs of *FTL_0304* infected mice at any of the time points examined (Fig. 6). These results demonstrate that the induction of an early pro-inflammatory cytokine response in mice immunized with the *FTL_0325* mutant is also associated with an early recruitment of inflammatory cells to the sites of infection in the lungs.

### Immunization with the *FTL_0325* or *FTL_0304* Mutants Induce Similar Humoral Immune Responses

**Figure 7:**
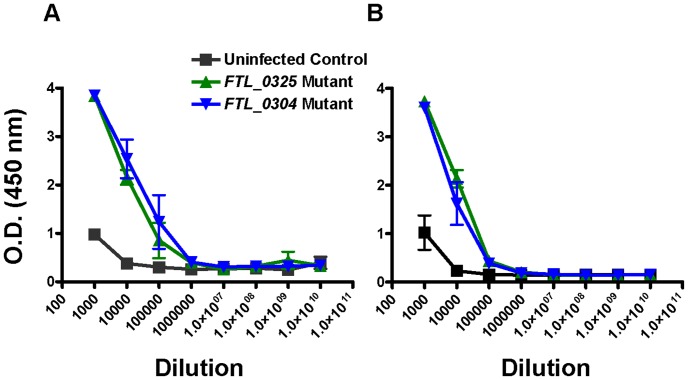
Antibody responses in mice immunized with the *FTL_0325* and *FTL_0304* mutants. BALB/c mice (n = 3) were immunized with 1×10^7^ CFU of the mutants intranasally. The mice were bled at days 45 (A) and 60 (B) post-immunization and total IgG levels were determined in the serum by ELISA.

It was observed that the mice immunized with the *FTL_0325* mutant were better protected against i.n. *F. tularensis* SchuS4 challenge than the *FTL_0304* immunized mice. Moreover, mice immunized with the *FTL_0325* mutant induced an early innate immune response as evidenced by the higher pro-inflammatory cytokines and an early establishment of inflammatory foci in the lungs of infected mice. Next, it was investigated if differences in the adaptive immune response also account for the better protective efficacy of the *FTL_0325* mutant. *F. tularensis* specific IgG levels were determined at days 45 and 60 post-immunization in the sera of immunized mice by ELISA. A significantly higher level of *F. tularensis* specific IgG antibodies were found in the sera of mice vaccinated either with the *FTL_0325* or *FTL_0304* mutant as compared to the naïve mice. However, no differences were observed in the titers of total IgG antibodies between mice immunized either with the *FTL_0325* or *FTL_0304* mutant (Fig. 7A and B). These results suggest that the humoral immune response may not be a significant contributing factor in the enhanced protection observed in the *FTL_0325* immunized mice against the *F. tularensis* SchuS4 challenge.

### Immunization with the *FTL_0325* Mutant Results in a Potent Memory Recall Response

**Figure 8:**
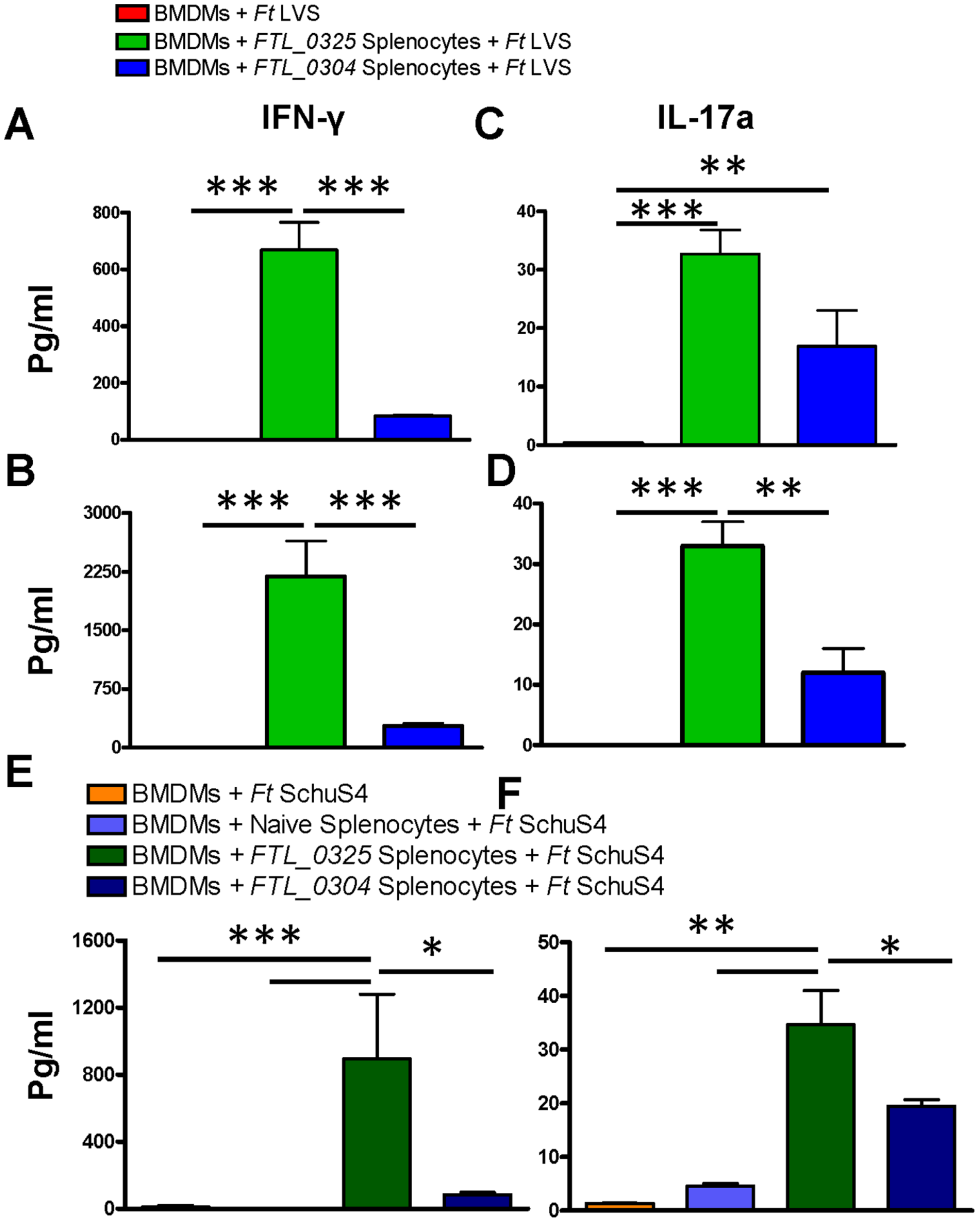
Immunization with the *FTL_0325* mutant results in a potent memory recall response. Cell culture supernatants collected from BMDMs infected with either the *F. tularensis* LVS (A–D) or *F. tularensis* SchuS4 (E and F) and co-cultured with the splenocytes prepared from immunized mice at day 45 post-immunization (A and C) or at day 60 (B–F) were analyzed for IFN-γ (A, B and E) and IL-17a (C,D and F) cytokines by Cytometric Bead Flex sets. The data are representative of two independent experiments and were analyzed using ANOVA with Tukey-Kramer Multiple Comparison post-test and *P* values recorded. *P<0.05; **P<0.01; ***P<0.001.

**Figure 9:**
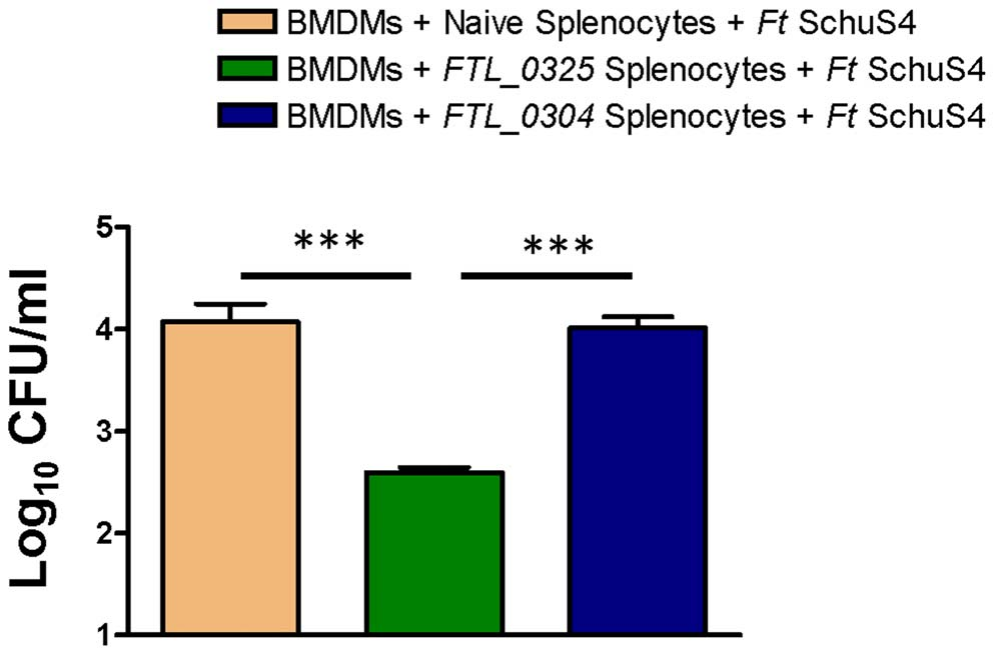
Recall memory response results in an enhanced killing of *F. tularensis* SchuS4. The BMDMs were infected with *F. tularensis* SchuS4 and co-incubated with the splenocytes from naïve, *FTL_0325* or *FTL_0304* immunized mice. The BMDMs were lysed 72 hrs PI, diluted 10-fold and plated on MH-chocolate agar plates to determine the intracellular bacterial replication. The results were expressed as log_10_ CFU/ml and are representative of two independent experiments. The data were analyzed using ANOVA with Tukey-Kramer Multiple Comparison post-test and *P* values were recorded. ***P<0.001.

It has recently been reported that the in vivo protective ability of a vaccine can also be evaluated by observing the expression of a select group of inflammatory mediators in an ex vivo assay [29]. We performed a splenocyte co-culture assay to establish the differences in the degree of protective efficacy observed between the mice vaccinated with the *FTL_0325* and *FTL_0304* mutants. BMDMs derived from naïve BALB/c mice were infected either with a 100 MOI of *F. tularensis* LVS or 20 MOI of *F. tularensis* SchuS4 for two hrs and then treated with gentamicin to kill all the extracellular bacteria. The infected BMDMs were co-cultured with splenocytes from mice immunized either with 1×10^7^ CFU of the *FTL_0325* or *FTL_0304* mutant isolated at day 45 and 60 post-immunization. The co-cultures were allowed to proceed for 48 hrs for *F. tularensis* LVS or for 120 hrs for *F. tularensis* SchuS4. The cell culture supernatants were collected and analyzed for IFN-γ and IL-17a cytokines. Significantly higher levels of IFN-γ were observed in BMDMs infected either with *F. tularensis* LVS (Fig. 8A and B) or SchuS4 (Fig. 8E) and co-cultured with splenocytes from *FTL_0325* immunized mice as compared to those observed for BMDMs co-cultured with splenocytes from *FTL_0304* immunized mice. A similarly elevated level of IL-17a was also observed in BMDMs infected either with *F. tularensis* LVS (Fig. 8C, D) or SchuS4 (Fig. 8F) and co-cultured with splenocytes from *FTL_0325* immunized mice. Concurrent with the cytokine responses, an enhanced killing of *F. tularensis* SchuS4 was also observed in BMDMs co-cultured with splenocytes from the *FTL_0325* immunized mice (Fig. 9). These results indicate that immunization with *FTL_0325* not only induces a higher degree of protection in the immunized mice but also generates an enhanced ex- vivo memory recall response at days 45 and 60 post-immunization.

## Discussion

One hundred years since the discovery of *F. tularensis*, attempts to develop an effective vaccine against tularemia have met with limited success. Subunit or killed vaccines provided limited protection against *F. tularensis* SchuS4 [6], [32]–[35]. Similarly, mutants of *F. tularensis* LVS due to over attenuation of virulence, either failed or conferred protection only when an i.d. challenge route was used. Attenuated mutants generated on SchuS4 background have shown promising results and protected immunized mice against the fully virulent *F. tularensis* strains irrespective of the route of immunization [24], [36], [22]. Although these SchuS4 mutants have the potential to be developed into live attenuated vaccines, Tier 1 Select Agent status of SchuS4 may pose serious regulatory issues for the use of these mutants for mass immunizations. Since aerosolization is the most feared route of spread of *F. tularensis* in a bioterror attack and based on previous observations barring two recent reports [24], [36] that i.d. immunizations induce suboptimal protection against respiratory challenge [11]–[14], in the present study i.n. immunization and challenge was preferred over the i.d. route. In this study, we describe a live attenuated vaccine candidate which is as efficacious as the SchuS4 mutants in its protective ability, along with possessing an additional advantage of being generated on the *F. tularensis* LVS background.

We demonstrate that all the four mutants were well tolerated by mice at a dose as high as 1×10^7^ CFU by i.n. route which represents a 1000 LD_100_ for the wild type *F. tularensis* LVS in our laboratory. No adverse clinical signs or death was observed and necropsy of the mice sacrificed at various times PI indicated no signs of gross pathological lesions in the lungs, liver or spleen (not shown). Three of the four mutants, *FTL_0325*, *FTL_0291* and *FTL_0057* retained their immunogenic potential despite an attenuation of virulence. A single dose i.n. immunization with the *FTL_0325*, *FTL_0291* and *FTL_0057* mutants provided 100% protection against i.n. SchuS4 challenge in BALB/c mice. An immunization regimen using ten-fold less immunization dose (1×10^6^ CFU, i.n.) did not alter the protective ability of these three mutant strains (data not shown) indicating that lower immunization doses may be equally effective in rendering protection. Interestingly, the *FTL_0304* mutant which was as attenuated for virulence as the other three mutants, failed to provide protection against *F. tularensis* SchuS4.

We have characterized and recently reported the role of *FTL_0325* and its ortholog *FTT0831c* in *F. tularensis* SchuS4 in intramacrophage survival and suppression of pro-inflammatory cytokines [25]. In this study, we demonstrate that the *FTL_0325* mutant in addition to its role in intramacrophage survival is similarly required for in vivo growth and virulence in mice. The results from this study demonstrate that although the *FTL_0325* mutant survives and disseminates to multiple organs in the host, it is incapable of causing a lethal infection. Additionally, as observed in macrophages [25], the loss of *FTL_0325* also results in the induction of pro-inflammatory cytokines as early as day 1 PI in the lungs of immunized mice. No correlation between bacterial numbers and cytokine responses at day 1 PI between *F. tularensis* LVS, the *FTL_0325* and *FTL_0304* mutant infected mice demonstrates that the observed differences in the levels of pro-inflammatory cytokines are not attributable to the differences in the bacterial load. In fact, these findings reflect the unique ability of the *FTL_0325* mutant to induce an early pro-inflammatory cytokine response. Taken together, these findings support our observations that mutation of *FTL_0325* leads to the loss of immune subversive properties of LVS under in vitro [25] as well as in vivo conditions. The increased pro-inflammatory cytokine response in the *FTL_0325* immunized mice was also associated with an early influx of neutrophils, establishment of inflammatory foci and bacterial clearance. These unique characteristics of the *FTL_0325* mutant meet the criteria for an ideal vaccine candidate for tularemia which as has been proposed, should lack immune-subversive properties and residual virulence of *F. tularensis* LVS [37].

The Na^+^/H^+^ antiporter encoded by the *FTL_0304* gene of *F. tularensis* is required for the homeostasis of Na^+^, H^+^ and other monovalent cations. It has been demonstrated for several bacterial pathogens that the loss of Na^+^/H^+^ antiporters results in increased sensitivity to pH, sodium, potassium, and lithium salts, attenuation of virulence and colonization in mice [38], [39]. The mutants of *F. novicida* [40] and *F. tularensis* SchuS4 [41] in Na^+^/H^+^ antiporter gene are attenuated for virulence in mice and hepatic cell line, respectively. Although, transcomplementation of the *FTL_0304* mutant was not performed in this study, it was observed that the transcription of genes up- and downstream of *FTL_0304* was not affected as tested by quantitative real-time PCR ruling out any polar effects due to the insertion of a transposon in the *FTL_0325* gene (data not shown). Our in vitro characterization results demonstrate that the *FTL_0304* mutant of *F. tularensis* LVS does not exhibit any growth defect under acellular growth conditions but is attenuated for intramacrophage growth in MH-S cells [25]. Further, the *FTL_0304* mutant does not show an increased sensitivity towards lithium chloride, ethidium bromide or potassium chloride; however, it is sensitive to sodium chloride (data not shown). The *FTL_0304* mutant appeared to be highly attenuated for virulence and significantly fewer numbers of this strain were recovered as compared to the *FTL_0325* mutant throughout the course of infection. In contrast to *FTL_0325*, a lower induction of pro-inflammatory cytokines was observed in the lungs and spleen of the *FTL_0304* mutant immunized mice. A positive correlation between the bacterial numbers and cytokine responses at day 4 PI indicates that the observed differences in cytokine responses between the *FTL_0325* and *FTL_0304* mutant infected mice may be a consequence of differences in the bacterial load of the organs. However, the reason for consistently lower pro-inflammatory cytokines in the *FTL_0304* immunized mice is not known and remains a subject of continued investigation in our laboratory. Of the other two mutants of *F. tularensis* LVS used in this study, the *FTL_0057* encodes for a conserved hypothetical membrane protein. This mutant has also been identified in the *F. novicida* genetic screen and shown to be attenuated for virulence in mice [40]. Another mutant in *FTL_0291* gene which encodes for an aromatic amino acid transporter has not been identified in any of the previous genetic screens. The roles of *FTL_0057* and *FTL_0291* in *F. tularensis* pathogenesis are not known. The results from this study demonstrate that *FTL_0057* and *FTL_0291* mutants of *F. tularensis* are attenuated for virulence in mice and provide protection against i.n. challenge with *F. tularensis* SchuS4 strain. A detailed investigation is warranted to examine the mechanism(s) of immune protection observed following immunization with these two mutants.

The observed differences in the protective ability of the *FTL_0325* and *FTL_0304* mutants strongly support the notion that in addition to an attenuation of virulence, the mutant strains should be able to persist in the host for a sufficient period of time for the induction of an effective immune response. Our in vivo studies show that the *FTL_0304* mutant is highly attenuated for virulence in mice and gets cleared rapidly from the lungs, liver and spleen of the infected mice. The *FTL_0304* mutant disseminates from the lungs to spleen at significantly lower numbers as compared to the *FTL_0325* mutant, indicating that the mutant does not survive well in the host. A high sensitivity to salt does indicate one potential reason for its low survival and dissemination in the host. Similar to the results obtained for the *FTL_0304* mutant in this study, several previous studies have also shown that highly attenuated mutants of the *F. tularensis* LVS carrying gene deletions of O-antigen, LPS, or the capsular capB genes, when used as vaccines did not protect BALB/c mice against an i.n. SchuS4 challenge [42]–[44]. In this study, significantly higher levels of *F. tularensis* specific IgG antibodies were produced in the mice vaccinated with the *FTL_0325* or *FTL_0304* mutant compared to the unvaccinated mice. The higher levels of *F. tularensis* specific antibodies however, fail to explain the observed differences in the protective abilities of the *FTL_0325* and *FTL_0304* mutants. These observations suggest that cellular immunity plays a more critical role than the antibody mediated responses to protect against a SchuS4 challenge in mice immunized with the *FTL_0325* mutant. It has been reported that cell-mediated immunity acts synergistically with the humoral immune responses to provide protection against the *Francisella* infection [45], [46].

It has been reported that following the clearance of *F. tularensis* LVS, the number of cells infiltrating the lungs of vaccinated mice gradually decline and become similar to that of naïve mice. Also, *Francisella* specific effector memory T-cells are not continuously recruited and maintained in the lungs of these mice [47]. Furthermore, the activities of the *F. tularensis* LVS primed lung and liver lymphocytes are similar to the splenocytes primed through a homologous route [47]. Based on these observations, we used splenocytes from the immunized mice to evaluate a memory recall response. Since both the *FTL_0325* and *FTL_0304* mutants get cleared from the lungs of immunized mice at days 14 and 7 respectively, and the mice immunized with *FTL_0325* mutant were fully protected from *F. tularensis* SchuS4 challenge at day 30 post-immunization, the recall responses were evaluated at days 45 and 60 post immunization to determine the duration of immunity. The hallmark of a successful vaccination is the generation of an effector memory response. Although we used the LVS based *FTL_0325* mutant as a vaccine, a potent recall response was observed both against the homologous *F. tularensis* LVS and a heterologous *F. tularensis* SchuS4 strain. Our recall response studies using the LVS reaffirm the previous observations that production of IFN-γ upon recall stimulation is a hallmark of memory response against *F. tularensis* [48], [49]. A similar observation using SchuS4 in our recall response studies indicate that IFN-γ and IL-17 responses also play an essential role in the protective immunity in the *FTL_0325* immunized mice and may serve as markers for a successful vaccination strategy. Concurrent with the cytokine responses, an enhanced killing of SchuS4 by BMDMs co-cultured with the splenocytes derived from the *FTL_0325* mutant immunized mice at 45 days-post immunization indicate that the immunity persists well beyond the 30 days post-challenge period used in this study. Elevated levels of IFN-γ and IL-17 upon challenge with *F. tularensis* SchuS4 in vaccinated mice have been shown to be associated with protection [50], [51]. It has further been reported that IL-17a-dependent macrophage derived IFN-γ activates the macrophages to control the *F. tularensis* infection [52]. Collectively, our results demonstrate that the recall response of splenocytes can clearly distinguish a protective and a non-protective immune response observed in the *FTL_0325* versus *FTL_0304* immunized mice against a *F. tularensis* SchuS4 challenge.

To conclude, we report that the *FTL_0304*, *FTL_0291, FTL_0325* and *FTL_0057* mutants are not only attenuated for virulence, but the latter three mutants also provide 100% protection against *F. tularensis* SchuS4 in immunized mice. The data from the present study clearly establish a link between the ability of mutants to persist and their inflammatory potential to their capacity to induce a protective immunity. The limited growth of *FTL_0325* mutant along with its unique ability to induce an early cytokine and inflammatory response in the lungs, perhaps indicates that the host defense mechanisms triggered by this strain not only mount an effective innate immune response to terminate the bacterial infection, they also induce a long-term protective immune response. On the contrary, the rapid clearance of the *FTL_0304* mutant probably due to its inability to survive in the host, results in a muted immune response insufficient to provide protection against a subsequent *F. tularensis* SchuS4 challenge. Collectively, this study describes a mutant in the *FTL_0325* gene of *F. tularensis* LVS as a live attenuated candidate which may be developed into an effective vaccine against tularemia and a deletion mutant of *FTL_0325* may prove useful for any potential clinical use.
